# Direct Reprogramming of Mouse Fibroblasts to Neural Stem Cells by Small Molecules

**DOI:** 10.1155/2016/4304916

**Published:** 2015-12-16

**Authors:** Yan-Chuang Han, Yoon Lim, Michael D. Duffieldl, Hua Li, Jia Liu, Nimshitha Pavathuparambil Abdul Manaph, Miao Yang, Damien J. Keating, Xin-Fu Zhou

**Affiliations:** ^1^School of Pharmacy and Medical Sciences, Sansom Institute, University of South Australia, Adelaide, SA 5000, Australia; ^2^Department of Human Physiology and Centre for Neuroscience, Flinders University of South Australia, P.O. Box 2100, Adelaide, SA 5001, Australia

## Abstract

Although it is possible to generate neural stem cells (NSC) from somatic cells by reprogramming technologies with transcription factors, clinical utilization of patient-specific NSC for the treatment of human diseases remains elusive. The risk hurdles are associated with viral transduction vectors induced mutagenesis, tumor formation from undifferentiated stem cells, and transcription factors-induced genomic instability. Here we describe a viral vector-free and more efficient method to induce mouse fibroblasts into NSC using small molecules. The small molecule-induced neural stem (SMINS) cells closely resemble NSC in morphology, gene expression patterns, self-renewal, excitability, and multipotency. Furthermore, the SMINS cells are able to differentiate into astrocytes, functional neurons, and oligodendrocytes *in vitro* and *in vivo*. Thus, we have established a novel way to efficiently induce neural stem cells (iNSC) from fibroblasts using only small molecules without altering the genome. Such chemical induction removes the risks associated with current techniques such as the use of viral vectors or the induction of oncogenic factors. This technique may, therefore, enable NSC to be utilized in various applications within clinical medicine.

## 1. Introduction

Recently, fibroblasts have been reprogrammed into induced neural stem cells (iNSC) by transcription factors [1–5], which makes the neural stem cell (NSC) therapy for neurodegenerative disease feasible. However, clinical utilization of patient-specific NSC for the treatment of human diseases remains elusive, mainly due to the risks associated with viral transduction vectors used for induction. Several studies have shown that some small molecules can directly modify epigenetics and improve somatic cell reprogramming by regulating signaling pathways. For example, valproic acid (VPA) inhibits histone deacetylase and improves the efficiency of reprogramming mouse embryonic fibroblasts (MEF) into induced pluripotent stem (iPS) cells [6]. RG108 is a DNA methyltransferase inhibitor, which improves the efficiency of MEF into iPS cells [7]. Vitamin C (VC) is a cofactor in reactions driven by dioxygenases including collagen prolyl hydroxylases, hypoxia-inducible factor (HIF), prolyl hydroxylases, and histone demethylases, which has been found to enhance the generation of mouse and human iPS [8]. BIX01294, a G9a HMTase inhibitor, has been found to improve the efficiency of cell reprogramming [9]. A83-01 strongly inhibits ALK4, 5, and 7 and only weakly inhibits ALK1, 2, 3, and 6 and appears to inhibit TGF-*β*-induced epithelial-to-mesenchymal transition via the inhibition of Smad2 phosphorylation [10]. CHIR99021 is an inhibitor of glycogen synthase kinase 3*β* (GSK3*β*) that prevents the phosphorylation of beta catenin by GSK3*β* and activates Wnt signaling [11, 12]. MEK inhibitor PD0325901 can inhibit the MAPK/ERK signaling pathway to promote mouse embryonic stem cell (ESC) self-renewal [11, 13, 14]. Furthermore, iPS cells were induced from mouse fibroblasts by eight small molecules without using any transcription factors [15].

NSC have a strong potential to repair neurodegenerative diseases and enhance the regeneration of the damaged nervous system [7, 16], however there is still not a viral transduction vector free method to obtain a sufficient number of NSC for individualized therapies. Here, we set out to determine whether using only small molecules, in place of potentially hazardous transduction vectors, could induce mouse fibroblasts into NSC.

## 2. Materials and Methods

The animal ethics had been approved by the Flinders University Animal Ethic Committee and South Australia Pathology Animal Ethic Committee.

### 2.1. Cell Culture

Mouse embryonic fibroblasts (MEF) and tail-tip fibroblasts (TTF) were isolated from C57/BL6 mice as described previously [17]. MEF and TTF were cultured in DMEM (Life Technologies) containing 10% FBS (Life Technologies), 50 units/mL penicillin, and 50 *μ*g/mL streptomycin (Life Technologies).

### 2.2. Induction of SMINS Cells

MEF or TTF were seeded at 1.4 × 10^5^ per 35 mm dish coated with feeder cells before induction. MEF (Passages 1–3) were treated with mitomycin C (10 *μ*g/mL) for 2.5 hours and then washed three times with 1 × PBS and finally cultured in stem cell culture medium overnight for feeder cells. The stem cell signaling pathway modulator small molecules PD0325901, CHIR99021, and A83-01 were used to start the induction. The epigenetic modulator small molecules valproic acid, Bix01294, and RG108 were selected to improve the induction efficiency and the cell senescence modulator small molecule vitamin C was used to reduce cell death during the induction [18]. The cells were induced in 6 cycles. On the first day, the cells were induced in stem cell culture medium (SCM) (DMEM supplemented with 15% FBS, 1% nonessential amino acids (Life Technologies), 1% L-glutamine (Life Technologies), 50 units/mL penicillin, 50 *μ*g/mL streptomycin, 0.1 mM *β*-mercaptoethanol (Life Technologies), and 1,000 units mL^−1^ leukaemia inhibitory factor (LIF) (Millipore)) containing small molecules (valproic acid, 1 *μ*M; Bix01294, 1 *μ*M; RG108, 0.04 *μ*M; PD0325901, 1 *μ*M; CHIR99021, 3 *μ*M; vitamin C, 25 *μ*M; A83-01, 2.5 *μ*M). The cells were cultured in SCM for the next two days. Then, the cycle was repeated 5 times. Next, the cells were passaged and suspended in 1 mL SCM (as for per 35 mm dish) and then did a drop of 20 *μ*L for suspending culture in petri dishes as shown in Supplementary Figure S1 available online at http://dx.doi.org/10.1155/2016/4304916. Finally, the cells were cultured in the neural stem cell medium (DMEM/F12 (Life Technologies) supplemented with B-27 (1 : 50, Life Technologies), 50 units/mL penicillin, 50 *μ*g/mL streptomycin, 8 mM HEPES buffer, 20 ng/mL EGF, and 10 ng/mL bFGF) in petri dishes for two weeks. As for feeder-free induction, the cells were seeded at 5 × 10^5^ cells per 35 mm dish coated with PDL (10 *μ*g/mL) (Sigma) at 37°C for two hours. The cells were cultured in SCM containing Bix01294, RG108, and PD0325901 for 2 weeks; the medium was changed on the other day. The colonies appeared during the induction process. The colonies were cultured in petri dishes in NSC medium for another two weeks. Native NSC were cultured from brain of new born mouse in the NSC medium as positive controls as described previously [19]. All the small molecules were from Stemgent.

### 2.3. RT-PCR and RT Profiler PCR Array

Total RNA was extracted using the RNeasy Mini Kit (Qiagen) with on-column DNA digestion. Total RNA (500 ng) was converted to cDNA by Superscript III Direct cDNA Synthesis System (Life Technologies). PCR was performed by 30 cycles using the primers described in Supplementary Table 3. The RT profiler PCR array was carried out using the Mouse Neurogenesis and NSC PCR Array (Qiagen).

### 2.4. Alkaline Phosphatase (ALP) and Immunofluorescence Staining

ES culture medium was added to NS and SMINS cells overnight. Alkaline phosphatase staining was carried out according to the manufacture's protocol (Roche). For the immunocytochemistry staining, cells were washed with 1 × PBS and then fixed with 4% paraformaldehyde for 10 min. After washing twice with 1 × PBS, cells were permeabilized with 0.1% Triton X-100 for 20 min. Cells were then washed twice and blocked in a solution of PBS containing 1% FBS and 4% BSA for 1 hour. Primary antibodies were diluted in blocking buffer and applied for 1 hour at room temperature or overnight at 4°C. Primary antibodies were used at the following dilution: Sox2 (Millipore, 1 : 200, mouse), Olig2 (Millipore, 1 : 500, rabbit), GFAP (Dako, 1 : 400, rabbit), Map2 (Osenses, 1 : 1000, rabbit), Nestin (Santa Cruz Biotechnology, 1 : 300, mouse), Oct4 (N-19) (Santa Cruz Biotechnology, 1 : 500, goat), Vamp2 (Osenses, 1 : 2000, rabbit), NeuN (Biosensis, 1 : 500, mouse), Alpha-tubulin (Sigma, 1 : 1000, mouse) and O4 (Millipore, 1 : 200, mouse), and Ki67 (Abcam, 1 : 100, rabbit). Cells were washed three times with 1 × PBS and then applied with secondary fluorescent antibodies (1 : 1000, Cy3 or Alexa-488) and 10 *μ*g/mL DAPI for 1 hour at room temperature.

### 2.5. FACS Analysis

TTF cells were dissociated and incubated in 2% FBS-PBS solution with antibody P75 conjugated with FITC (Biosensis, 1 : 6, mouse) on ice for a half hour. The cells were washed three times with ice-cold 2% FBS-PBS before running FACS. The positive fraction was evaluated by FACS (Beckman Coulter Epics Altra HyperSort, using Expo MultiComp Software version 1.2B (Beckman Coulter, Miami, FL, USA)) comparing with a blank control.

### 2.6. *In Vitro* Differentiation of SMINS Cells

Cells were seeded at 0.5 × 10^4^ on a PDL (10 *μ*g/mL)/laminin (10 *μ*g/mL) (Sigma) coated 4-well plate. For spontaneous differentiation, cells were cultured in NS cell culture medium containing N2 (Life Technologies) without EGF and bFGF for one or three weeks. For the differentiation of mature neuron, the single SMINS cells were cultured in neurobasal medium (Life Technologies) containing B27 (2%) (Life Technologies), GlutaMAX (2 mM) (Life Technologies), and dibutyryl cAMP (0.5 mM) (Sigma) for four weeks. As for specific astrocyte differentiation, the cells were cultured in neurobasal medium containing 1 × N2, 1 × B27, and 1% FBS for 3 weeks. For neuron differentiation, the cells were cultured in neurobasal medium containing 1 × N2, 1 × B27, 1% FBS, 5 *μ*M forskolin, and 1 mM retinoic acid for 2 weeks and then in neurobasal medium containing 1 × N2, 1 × B27, 1% FBS, 10 ng/mL BNDF, and 10 ng/mL GDNF for 2 weeks. The cells were cultured in DMEM/F12 containing 1 × N2, 10 ng/mL bFGF, 10 ng/mL PDGF-AA, and 5 *μ*M forskolin for 5 days and then in 0.2 mM vitamin C and 30 ng/mL T3 for 3 weeks for specific oligodendrocyte differentiation.

### 2.7. Differentiation of SMINS Cells* In Vivo*

Dissociated SMINS cells were labeled with lentiviral EGFP vectors and a total volume of 3 *μ*L (10^5^/*μ*L) was injected into the lateral ventricle of brain in nude pups (6 pups) at the age of 3 days. Brains were collected at 6 weeks after injection following a saline perfusion, fixed in periodate-lysine-paraformaldehyde for 24 hours [20], washed with PBS, soaked in 30% sucrose for 48 hours, and sectioned into 30 *μ*m coronal sections. Brain sections were immunostained for Sox2 (for neural stem cells), ki67 (for proliferating cells), GFAP (for astrocytes), Map2 (for neurons), NeuN (for neurons), and Olig2 (for oligodendrocytes) using our methods described previously [21].

### 2.8. Electrophysiology

Whole cell patch clamp was performed on differentiated cells using a HEKA EPC-10 patch clamp amplifier and Patch Master software (HEKA Electronik, Lambrecht/Pfalz, Germany). Patch pipettes were pulled from borosilicate glass and fire polished, with resistance of 3–5 MΩ. Internal solution contained the following (mM): NaCl, 10; KCl, 145; HEPES, 10; MgCl_2_, 1; and EGTA, 1, adjusted to pH 7.3. External solution contained the following (mM): NaCl, 135; KCl, 2.8; HEPES, 10; MgCl_2_, 1; CaCl_2_, 2; and Glucose, 10, adjusted to pH 7.4 with NaOH. Measurement of Na^+^ and K^+^ currents was performed in voltage-clamp mode, utilising a protocol with voltage steps of −70 to +70 mV (10 mV increments), for 20 ms or 100 ms, from a holding potential of −80 mV. Series resistance was compensated at least 70%. Action potentials were recorded in current-clamp mode, with injection of 20–50 pA of current if required. Voltages shown were not adjusted for liquid junction potential.

## 3. Results

### 3.1. Small Molecule-Induced Neural Stem (SMINS) Cells Can Be Obtained from MEF by a Combination of 7 Small Molecules

We selected a number of candidate small molecules to reprogram fibroblasts into NSC. A combination of small molecules (valproic acid, 1 *μ*M; Bix01294, 1 *μ*M; RG108, 0.04 *μ*M; PD0325901, 1 *μ*M; CHIR9901, 3 *μ*M; vitamin C, 25 *μ*M; A83-01, 2.5 *μ*M) is found to induce mouse embryonic fibroblasts (MEF) into NSC. Considering that too much expression of transcription factors is detrimental to the self-renewal of pluripotent cells [17], we designed a 6-cycle protocol for the induction process (Supplementary Figure S1). Fibroblasts were cultured alternatively in small molecule-containing stem cell culture medium (SMSCM) for 1 day and in stem cell culture medium (SCM) without small molecules for 2 days as cycle 1 and the cycle was repeated for additional 5 times. After the 6th cycle, the cells were cultured in suspension for 2 days and then in NSC culture medium for 2 weeks. There are no colonies in the induction process before suspending culture. There is one colony in each drop after suspending culture. And then the colonies were cultured in NSC medium for two weeks. MEF were negative for Sox2, Nestin, and SSEA-1 after several passages (Supplementary Figure S2). In order to eliminate the possibility of neural crest stem cells from mouse skin [22], only MEF that are negative to Sox2, SSEA-1, and Nestin were used for induction. Using 7 small molecules for induction, SMINS (SMINS-MEF-7) cells were able to be stably and homogenously expanded more than two years without a significant reduction in the self-renewal capacity and are morphologically indistinguishable from classic NSC either suspending culture in petri dishes or attaching on poly-D-lysine (10 *μ*g/mL)/laminin (10 *μ*g/mL) or matrigel coated cell culture dishes at high density (1 × 10^5^/cm^2^) (Supplementary Figures S3A and B and Figure S4). Firstly, we stained the colonies with ALP, and they appeared positive (Supplementary Figure S5). And then we tested the typical NSC markers Sox2 and Nestin; they were also positive (Figures S3C and D).

Next we tested the expression of NSC marker genes by the reverse transcription PCR (RT-PCR). Compared to fibroblasts, SMINS-MEF-7 cells expressed NSC marker genes including* Sox2*,* GFAP,* and* Olig2 *(Supplementary Figure S3E). Just like NSC, SMINS-MEF-7 cells did not express the pluripotent genes* Oct4* and* Nanog *(Figure S3E). In order to further assess the expression profiles of genes relevant to NSC, we carried out an analysis of 84 genes which are related to mouse neurogenesis and NSC utilizing RT profiler PCR arrays. Compared with MEF, 23 genes were upregulated by 3- to 1543-fold and 13 genes downregulated by at least 3-fold in SMINS-MEF-7 cells (Figure S6A and Supplementary Table 1). Notch [23, 24], Wnt [25], BMP [26, 27], and Shh [28] signaling pathways are known to regulate NSC properties. Among the upregulated genes,* Dll1*,* Notch2*,* Hey1,* and* Pou3f3* are involved in the Notch signaling pathway,* Shh* in the Shh signaling pathway, and* Bmp2* and* Bmp15* in the BMP signaling pathway. Among the downregulated genes,* Hey2* and* Heyl *are involved in the Notch signaling pathway,* Nog *in the BMP signaling pathway, and* Ndp* in the Wnt signaling pathway. Ten genes including* Notch2*,* Shh,* and* Fgf2* in SMINS-MEF-7 were upregulated in comparison with native NSC (Figure S6B and Supplementary Table 2).

To confirm the multipotency of the SMINS cells, we performed* in vitro* differentiation assays. SMINS-MEF-7 cells were able to spontaneously differentiate into astrocytes (GFAP-positive cells, 20 ± 2%), neurons (Map2-positive cells, 31 ± 3%), or oligodendrocytes (O4-positive cells, 36 ± 1%) (Supplementary Figures S7, 8, and 4). Moreover, SMINS-MEF-7 cells were able to express mature neural markers VAMP2 and NeuN in mature neuron differentiation medium (Figure S7). These results indicate that, like native NSC, SMINS cells are multipotent* in vitro*.

### 3.2. SMINS Cells Can Be Obtained from Tail-Tip Fibroblasts (TTF) by a Combination of 3 Small Molecules

**Figure 1:**
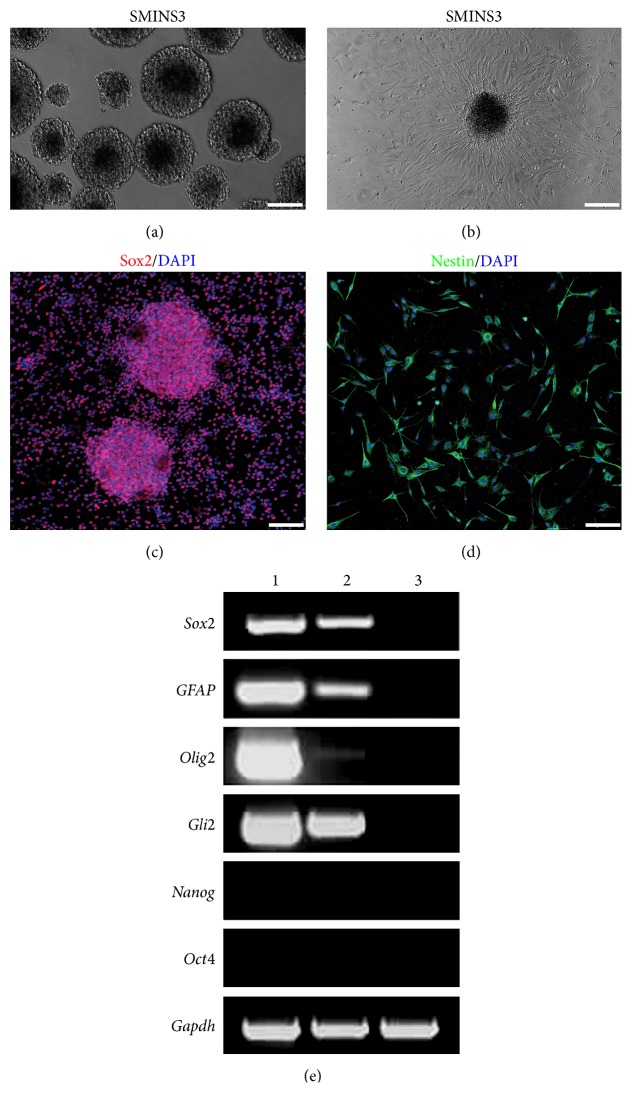
SMINS-TTF-3 cells induced from mouse tail-tip fibroblasts utilizing three small molecules (Bix01294, RG108, and PD0325901). (a) SMINS-TTF-3 neurospheres were cultured in petri dishes under bright field in suspending culture. (b) A SMINS-TTF-3 neurosphere was cultured on matrigel coated dishes under bright field in attaching culture. (c-d) SMINS-TTF-3 neurospheres were dissociated and stained by typical neural stem cell markers Sox2 (red) and Nestin (red) examined by immunocytochemistry. DAPI was used for nuclei counterstaining (blue). (e) Analysis of typical neural stem cell gene expressions by RT-PCR, (1) NS (native neural stem cells), (2) SMINS-TTF-3 (small molecule-induced neural stem cells from TTF with 3 small molecules), and (3) TTF (tail-tip fibroblasts). Scale bar: 100 *μ*m.

**Figure 2:**
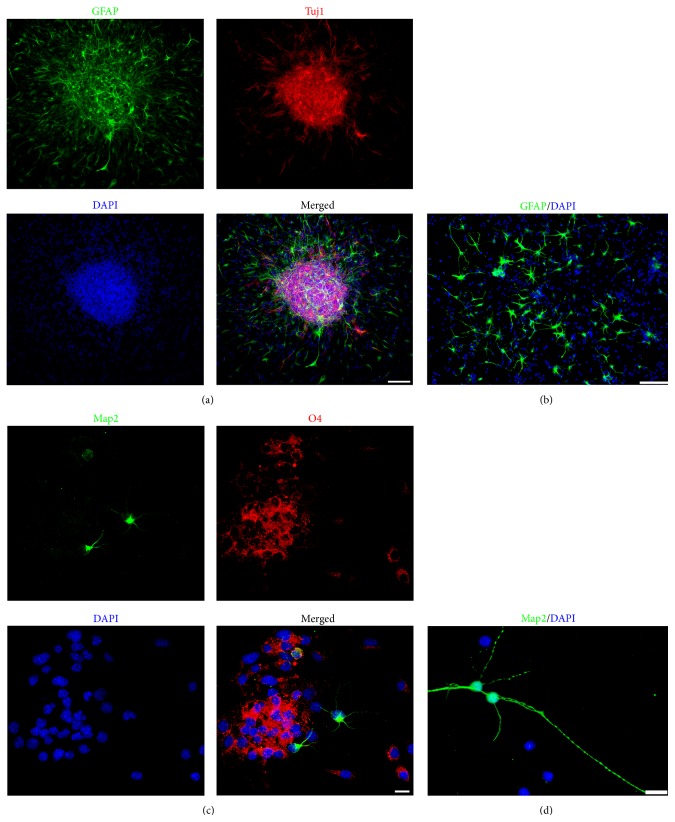
Differentiation of SMINS-TTF-3 cells* in vitro*. (a-b) SMINS-TTF-3 cells spontaneously differentiated into astrocytes marked by GFAP (green) and neurons marked by Tuj1 (red) in spontaneous medium for one week. (c-d) SMINS-TTF-3 cells spontaneously differentiate into neurons marked by Map2 (green) and oligodendrocytes marked by O4 (red) in spontaneous medium for three weeks. DAPI was used for nuclei counterstaining (blue). Scale bar: 100 *μ*m (a and b) and 10 *μ*m (c and d).

**Figure 3:**
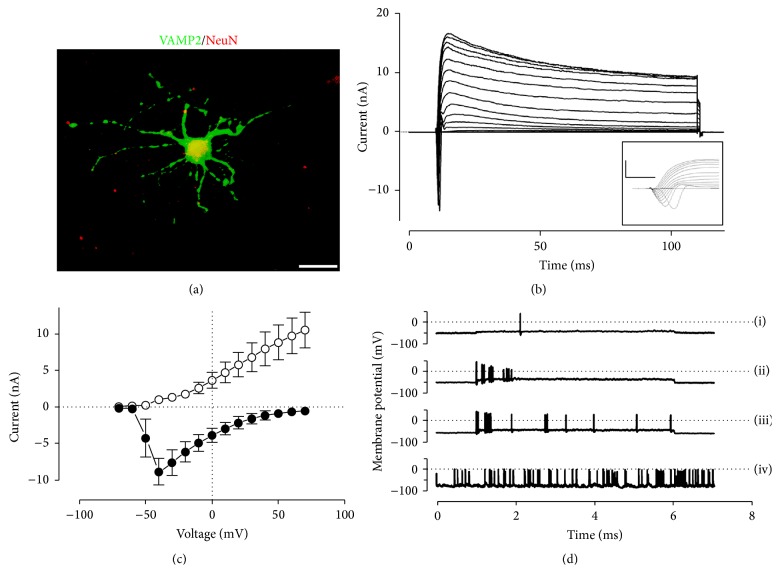
Mature neurons from SMINS-TTF-3 cells. (a) SMINS-TTF-3 cells were cultured in mature neural medium for one month and stained by mature neuron markers VAMP2 (green) and NeuN (Red). (b) Electrophysiological investigations of a subset of long-term differentiated cells showed the presence of both inward Na^+^ currents and outward K^+^ currents in response to electrical stimulation with steps from −70 to +70 mV (10 mV increments) from a holding potential of −80 mV. Representative trace with 100 ms steps, inset with 20 ms steps (inset scale bars represent 5 ms on *x*-axis, 10 nA on *y*-axis). (c) Mean ± SEM maximal Na^+^ (closed circles) and K^+^ currents (open circles, *n* = 4). (d) These cells demonstrated action potential firing in response to current injection ((i) 20 pA for 5 s, (ii) and (iii) 50 pA for 5 s) or spontaneously (iv). Scale bar: 10 *μ*m (a).

Next we examined which small molecules are important for the generation of SMINS cells by withdrawal of individual small molecules from the combination. We found that the small molecules Bix01294, RG108, and PD0325901 are important for the induction to occur. To further confirm the validity of the protocol to obtain SMINS cells from fibroblasts and to eliminate potential contamination from skin-derived neural crest stem cells, we isolated TTF from adult mouse tails which had been stripped of skin. In order to further eliminate the possible contamination of the neural crest cells in TTF, TTF were sorted by FACS with fluorescence-labelled antibody to p75. Only 0.1% TTF cells are p75-positive cells after 3 passages (Supplementary Figure S9). Only p75-negative TTF cells were used for induction. Just like MEF, TTF could also robustly form neurospheres after the 6 cycles' induction protocol with these three small molecules, Bix01294, RG108, and PD0325901. These SMINS (SMINS-TTF-3) cells resemble native NSC in morphology (Figures 1(a) and 1(b)). SMINS-TTF-3 cells also express the NSC markers Sox2, Nestin, and ALP (Figures 1(c)-1(d) and Supplementary Figure S5). Next we tested the expression of NSC genes by reverse transcription PCR (RT-PCR). SMINS-TTF-3 cells expressed NSC marker genes including* Sox2*,* GFAP*,* Olig2,* and* Gli2 *(Figure 1(e)) compared to fibroblasts which did not show this expression. Similar to NSC, SMINS-TTF-3 cells did not express the pluripotent genes* Oct4* and* Nanog *(Figure 1(e)). Furthermore, SMINS3 cells did not show pluripotent marker Oct4 by ICC (Supplementary Figure S10). Finally, we performed* in vitro* differentiation assays. SMINS-TTF-3 cells were able to differentiate into astrocytes (GFAP-positive cells, 24 ± 1%), neurons (Map2-positive cells, 36 ± 2%), or oligodendrocytes (O4-positive cells, 30 ± 2%) (Figure 2). Furthermore, SMINS-TTF-3 cells were able to express mature neuronal markers Vamp2 and NeuN in mature neuron differentiation medium (Figure 3(a)). To check whether the SMINS cells contain feeder cells, the SMINS cells after passage 5 were stained for fibroblast marker Alpha-tubulin. We did not find any Alpha-tubulin positive cells in the SMINS cells (Supplementary Figure S11), suggesting that there was no feeder cell contamination in SMINS cells after induction.

### 3.3. SMINS Cells Can Differentiate into Functional Neurons

Next, we checked whether the SMINS cells can differentiate into functional neurons. The differentiated SMINS-TTF-3 cells display positive mature neuron markers (Figure 3(a)). Furthermore, a small subset of differentiated SMINS3 cells displays a unique phenotype similar to that of mature neurons. Electrophysiological analysis demonstrated a resting membrane potential of −57.7 ± 5.2 mV (*n* = 5) in these cells which contained fast inactivating inward Na^+^ currents in addition to slowly inactivating outward K^+^ currents (Figures 3(b) and 3(c)). Action potentials either were spontaneous or were able to be evoked in these cells by injecting current pulses injection (Figure 3(d)). The majority of neural-like differentiated cells displayed a different phenotype, with a more positive resting membrane potential, only K^+^-like outward currents with no inward Na^+^ currents or evoked action potentials (Supplementary Figure S12). This indicates that the SMINS cells are able to differentiate into functional neurons.

### 3.4. SMINS Cells Can Differentiate to Neural Cell Lineages* In Vivo*

**Figure 4:**
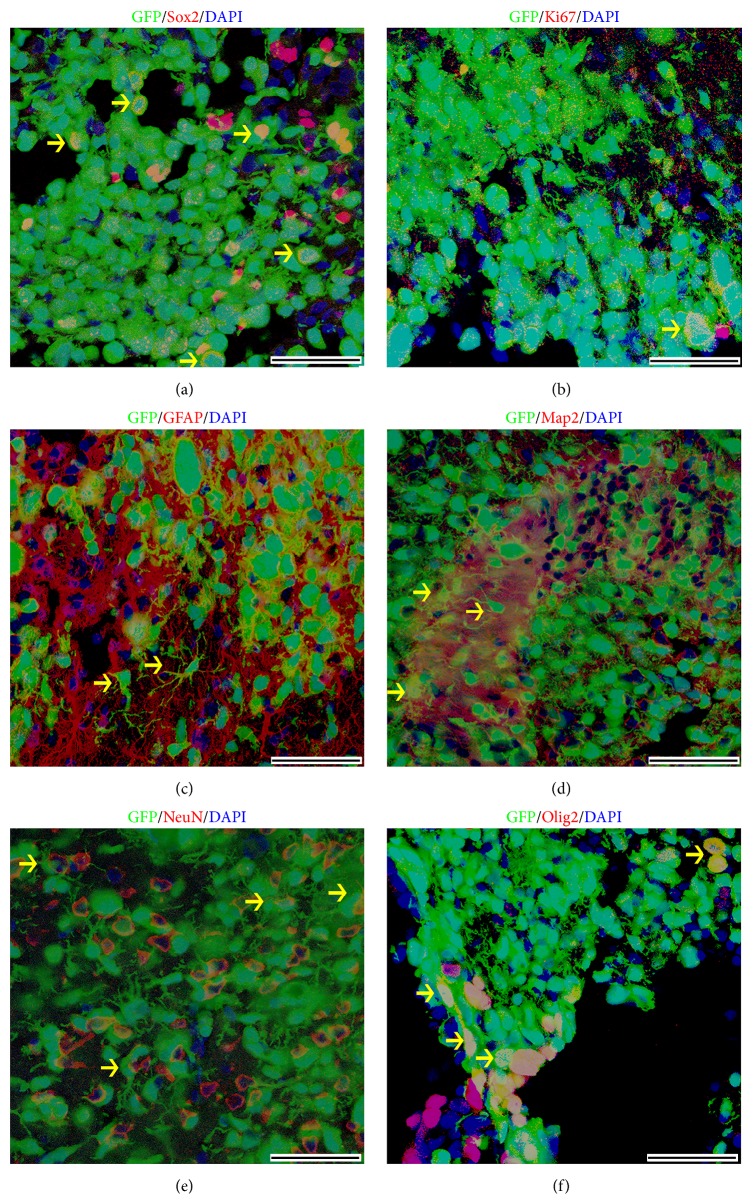
Differentiation of SMINS-TTF-3 cells* in vivo*. SMINS-TTF-3 cells infected with lentiviral EGFP vectors were injected into the lateral ventricle of brain in nude pups at the age of 3 days and the brains were collected at 6-week point. (a-b) Some injected SMINS-TTF-3 cells remained as neural stem cells, as indicated by neural stem cell marker Sox2/GFP^+^. Some cells kept the ability of proliferation, as shown by Ki67/GFP^+^ staining. (c–f) The injected cells differentiated into astrocytes (GFAP/GFP^+^), neurons (Map2/GFP^+^ and NeuN/GFP^+^), and oligodendrocytes (Olig2/GFP^+^)* in vivo*. DAPI was used for nuclei counterstaining (blue). Scale bar: 50 *μ*m. The arrows direct positive cells.

To assess whether the SMINS cells are able to survive and differentiate into neural cell lineages* in vivo*, SMINS-TTF-3 cells were labeled with green fluorescence protein (GFP) with lentiviral EGFP vector and were transplanted into lateral ventricle of nude pups. Only few GFP^+^ cells were Sox2 (Figure 4(a)) and Ki67 (Figure 4(b)) positive at 6 weeks after transplantation, which means almost all of SMINS cells differentiate to neural cell lineages* in vivo*. The GFP^+^ cells migrated with a long distance from lateral ventricles into the parenchyma and were well integrated with the host brain tissues (Supplementary Figure S12). Furthermore, the SMINS cells were positive to astrocyte marker GFAP (Figure 4(c)), neural markers Map2 and NeuN (Figures 4(d) and 4(e)), and oligodendrocyte marker Olig2 (Figure 4(f)). These data indicate that the SMINS cells are able to differentiate to neural cell lineages* in vivo*.

### 3.5. Induction Efficiency

It is difficult to calculate the induction efficiency because of the special induction process. No colony appeared before the drop suspending culture. Each drop formed one colony after suspending drop culture. So we calculated how many cells could form one colony per drop. We found the minimum cell number is 50 to form one colony. Therefore, the efficiency of induction is up to 2%. The induction efficiency of 2% is a relative number compared with suspending culture cells as described in the method section. A better way for the induction efficiency is to use of Sox2-EGFP fibroblasts for induction in the future.

### 3.6. Feeder-Free SMINS3 (FF SMINS3)

**Figure 5:**
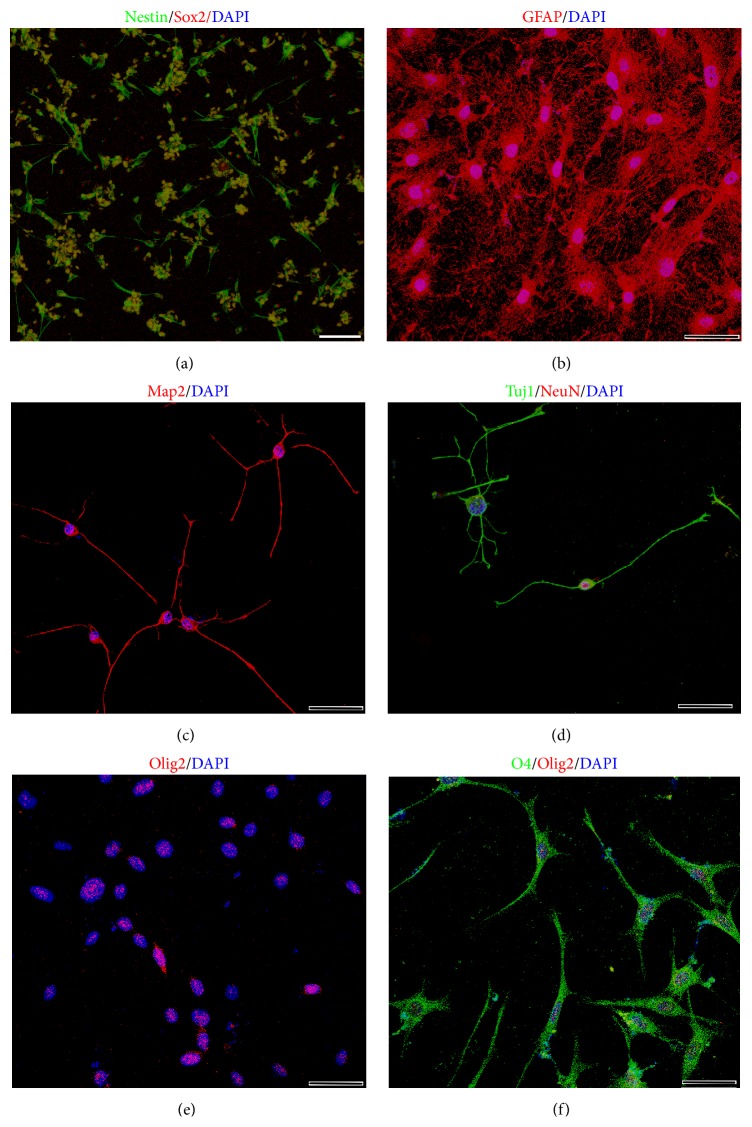
Feeder-free SMINS-TTF-3 cells (FF SMINS3). TTF were seeded on PDL coated dishes in stem cell medium containing Bix01294, RG108, and PD0325901 for two weeks and then transferred into NSC medium in petri dishes for two weeks. (a) The FF SMINS3 cells were stained by neural stem cell markers Sox2 and Nestin. (b–f) The FF SMINS3 cells differentiated to astrocytes (GFAP), neurons (Tuj1, Map2, and NeuN), and oligodendrocytes (Olig2 and O4) in specific differentiation medium. DAPI was used for nuclei counterstaining (blue). Scale bar: 100 *μ*m (a) and 50 *μ*m (b–f).

Our SMINS cells contain feeder cells in the first several passages, which can affect the NSC application in the future. So we tried to remove the feeder cells with some dish substrates. It was found that the poly-D-lysine (PDL) could replace feeder cells during the induction. Moreover, the induced cells formed colonies during the induction process on PDL, so the feeder free protocol does not need to perform suspending drop culture for the colony formation. The feeder-free SMINS3 were positive for NSC markers Nestin and Sox2 (Figure 5(a)). Furthermore, the cells were able to differentiate into astrocytes (Figure 5(b)), neurons (Figures 5(c) and 5(d)), and oligodendrocytes (Figure 5(e)) in specific differentiation medium. All these data indicate that the feeder-free SMINS3 are NSC.

## 4. Discussion

Reprogramming somatic cells to iNSC makes the NSC therapy feasible. iNSC also have great value as models of disease pathogenesis, drug screening, and toxicity tests. Although NSC can be generated from ESCs or iPS [29, 30], there are still ethic and safety problems when these cells are used for cell therapy in patients [30–32]. To overcome these problems, some scientists have successfully induced somatic cells to iNSC by overexpressing transcription factors [1–5]. However, they all used viral vectors to introduce transcription factor genes into the host cells, which brings the safety concern on the NSC therapy. Moreover, the c-Myc oncogene can cause brain tumorigenesis from transplanted iPS-derived NS cells [4]. Our study demonstrates that mouse fibroblasts can be efficiently induced into NSC using only small molecules. This is the first report that multipotent stem cells can be induced from fibroblasts without using any exogenous transcription factors. In our experiment, the SMINS cells were passaged for more than two years and still continue proliferating in either attaching or suspending culture. Thus, the SMINS cells are able to form stable cell lines for stem cell therapy. Our studies have made an important step forward towards tailoring individualized therapies for patients with neurodegenerative diseases and other neurological disorders, as our method eliminates the concerns of potentially harmful genome integration by viral transduction vectors or the introduction of oncogenic transcription factors. Thus, these SMINS cells may have a direct potential in clinical treatment of neurological disorders.

One issue that is concerned is the origin source of SMINS cells from MEF and TTF. We used two methods to eliminate possible neural crest contamination. By FACS method, only 0.1% TTF cells after 3 passages are p75-positive. These positive cells are most likely derived from p75-positive Schwann cells or blood vessel cells such as endothelial cells and smooth muscle cells [33]. Secondly, skin-derived neural crest cells were eliminated by stripping off the skin before the TTF preparation with enzymatic digestion. As the induction efficiency is 2%, it is unlikely that the SMINS cells are from the p75-positive neural crest cells, which only occupied 0.1% and was not used for the induction after FACS. Taken together, these results do not support the assumption of neural crest cells as the original source of the SMINS cells. Another issue to be considered is whether the SMINS cells pass by the pluripotent stage. Based on the present data, Oct4 and Nanog expression could not be detected in SMINS cells. Therefore, our data does not support the notion. However, we speculate that the SMINS cells may come through a partial pluripotent stage and become NSC when they were cultured in the NSC culture medium.

Although the mechanism of reprogramming is still unknown, it is related to DNA demethylation, histone demethylation, and acetylation [34–38]. The small molecules, such as VPA (histone deacetylase inhibitor), BIX01294 (G9a HMTase inhibitor), and RG108 (DNA methyltransferase inhibitor), can enhance reprogramming. It is reasonable to reprogram fibroblasts to NSC by these small molecules in proper conditions. It is reported that MEK inhibitor PD0325901 can inhibit the MAPK/ERK signaling pathway to promote mouse ESC self-renewal [11, 13, 14]. Our data also support the report that mouse pluripotent stem cells were differentiated to neuroectoderm by blocking MAPK/ERK signaling pathway [39]. Furthermore, our studies suggest that signaling pathways such as Notch, Shh, BMP, and Wnt are likely involved in the reprogramming of fibroblasts by these small molecules. It will be valuable in the future to understand how the small molecules affect each of the Notch, Shh, BMP, and Wnt pathways. SMINS cells may also provide a novel model for studying the mechanisms of reprogramming of somatic cells into adult stem cells. Moreover, it still remains unknown whether these small molecules could induce human fibroblasts to NSC.

## Supplementary Material

Supplementary Figure S1. A schematic diagram depicting the protocol for the production of small molecule-induced neural stem (SMINS) cells.
Supplementary Figure S2. MEF tested by neural stem cell markers.
Supplementary Figure S3. SMINS-MEF-7 cells induced from mouse embryonic fibroblasts.
Supplementary Figure S4. The positive control native neural stem cells (NS).
Supplementary Figure S5. The staining of Alkaline Phosphatase (ALP) for NS, SMINS and Fibroblasts.
Supplementary Figure S6. Quantitative analysis of 84 neural stem cell genes by RT profiler PCR arrays.
Supplementary Figure S7. Differentiation of SMINS-MEF-7 cells in vitro.
Supplementary Figure S8. Immunocytochemistry staining of fibroblasts using nervous cell markers.
Supplementary Figure S9. The sorting of negative TTF.
Supplementary Figure S10. SMINS3 cells were check by pluripotent stem cells marker.
Supplementary Figure S11. Immunocytochemistry staining of fibroblasts and SMINS cells using a fibroblast marker.
Supplementary Figure S12. Migration of SMINS-TTF-3 cells in vivo.
Supplementary Figure S13. The majority of differentiated cells demonstrate K+ currents only.
Supplementary Table S1. Analyses results of up-regulated and down-regulated genes for SMINS-MEF-7 cells (test) vs MEF (control).
Supplementary Table S2. Analyses results of up-regulated genes for SMINS-MEF-7 cells (test) vs NS (control).
Supplementary Table S3. The primers of RT-PCR in the experiment.
