# Supplementary material for: Direct Reprogramming of Mouse Fibroblasts to Neural Stem Cells by Small Molecules
Source: Stem Cells Int. 2015 Dec 16;2016:4304916. doi: 10.1155/2016/4304916 (PMC4695670; doi:10.1155/2016/4304916)
Supplement: Supplementary file 1 — Supplementary Figure S1. A schematic diagram depicting the protocol for the production of small molecule-induced neural stem (SMINS) cells. Supplementary Figure S2. MEF tested by neural stem cell markers. Supplementary Figure S3. SMINS-MEF-7 cells induced from mouse embryonic fibroblasts. Supplementary Figure S4. The positive control native neural stem cells (NS). Supplementary Figure S5. The staining of Alkaline Phosphatase (ALP) for NS, SMINS and Fibroblasts. Supplementary Figure S6. Quantitative analysis of 84 neural stem cell genes by RT profiler PCR arrays. Supplementary Figure S7. Differentiation of SMINS-MEF-7 cells in vitro. Supplementary Figure S8. Immunocytochemistry staining of fibroblasts using nervous cell markers. Supplementary Figure S9. The sorting of negative TTF. Supplementary Figure S10. SMINS3 cells were check by pluripotent stem cells marker. Supplementary Figure S11. Immunocytochemistry staining of fibroblasts and SMINS cells using a fibroblast marker. Supplementary Figure S12. Migration of SMINS-TTF-3 cells in vivo. Supplementary Figure S13. The majority of differentiated cells demonstrate K+ currents only. Supplementary Table S1. Analyses results of up-regulated and down-regulated genes for SMINS-MEF-7 cells (test) vs MEF (control). Supplementary Table S2. Analyses results of up-regulated genes for SMINS-MEF-7 cells (test) vs NS (control). Supplementary Table S3. The primers of RT-PCR in the experiment. [file 4304916.f1.zip › 4304916.f1/4304916.f1.pdf]

Supplemental Data

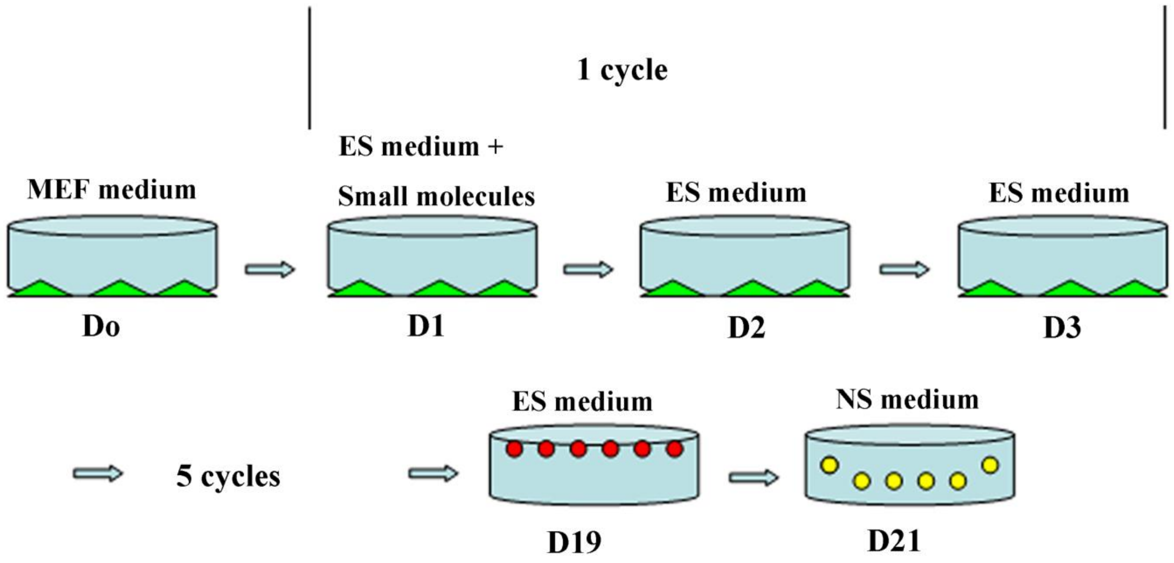

Supplementary Figure S1. A schematic diagram depicting the protocol for the production of small molecule-induced neural stem (SMINS) cells.

MEF or TTF were seeded at 1.4×10^5 per 35 mm dishes coated with feeder cells before induction. The cells were induced in 6 cycles. On the first day, the cells were induced in stem cell culture medium (SCM) containing small molecules. The cells were cultured in SCM for the next two days. Then, the cycle was repeated 5 times. Next, the cells were passaged and suspended in a drop of 20 µl SCM for two days. Finally, the cells were cultured in the neural stem cell medium by suspending culture for two weeks.

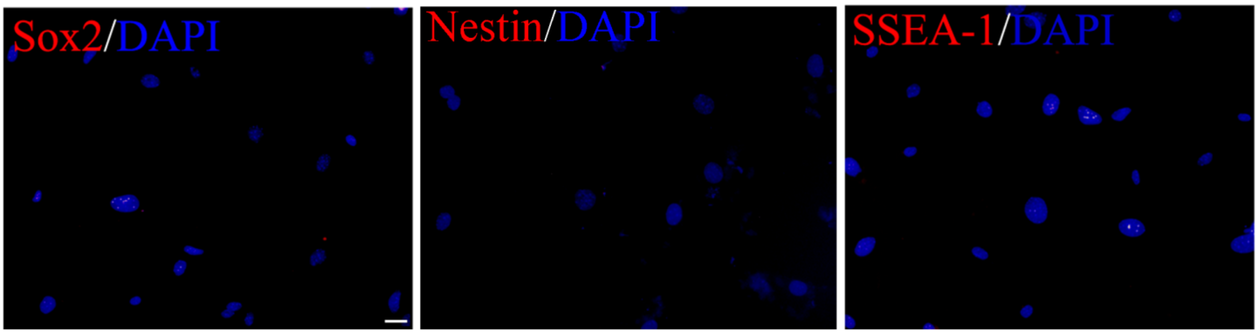

Supplementary Figure S2. MEF tested by neural stem cell markers.

MEF were stained by neural stem cell markers Sox2 (red), Nestin (red) and SSEA-1 (red). DAPI was used for nuclei counter-staining (blue). Scale bar: 10 μm.

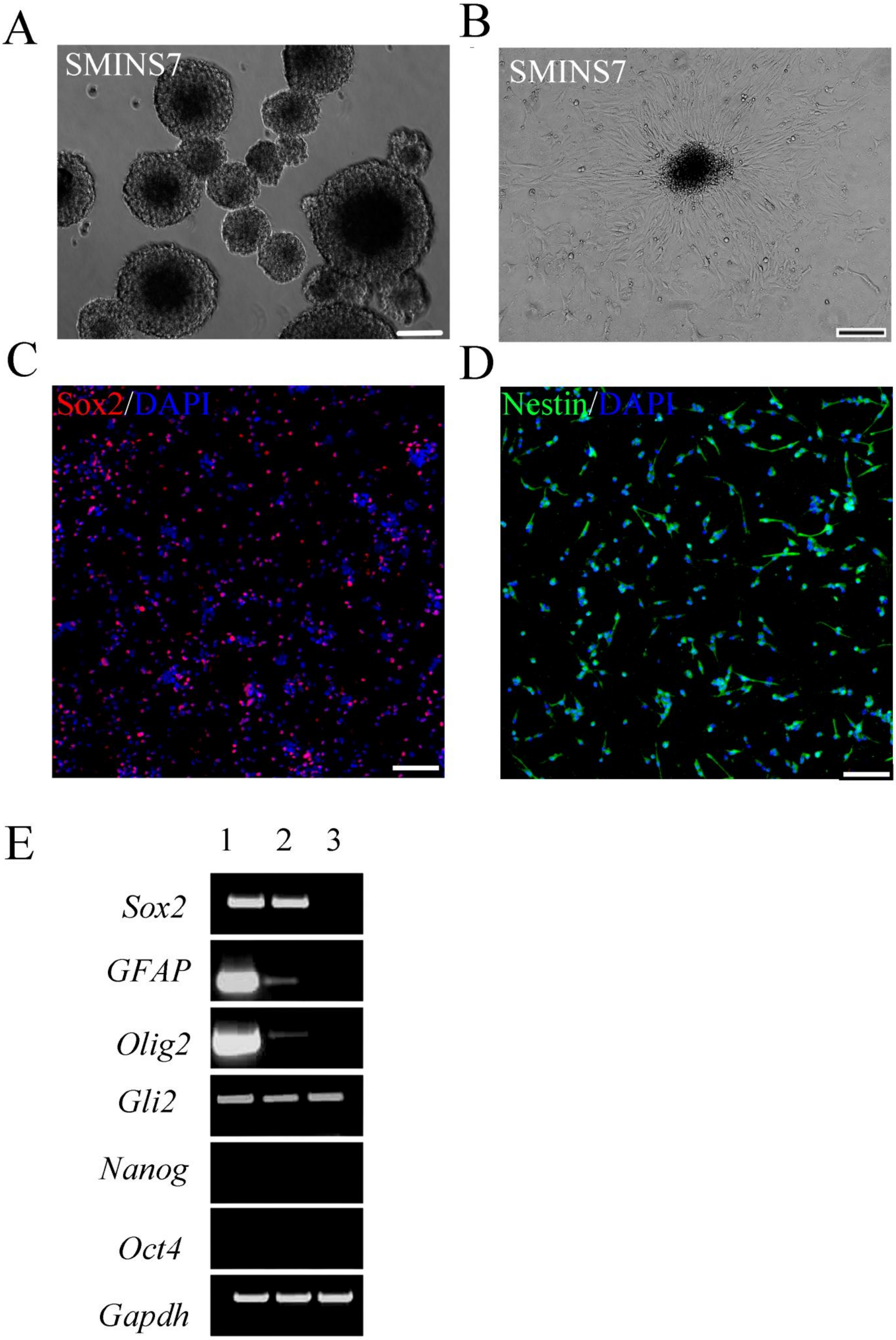
A
SMINS7
B
SMINS7
C
Sox2/DAPI
D
Nestin/DAPI
E
1
2
3
Sox2
GFAP
Olig2
Gli2
Nanog
Oct4
Gapdh

Supplementary Figure S3. SMINS-MEF-7 cells induced from mouse embryonic fibroblasts.

(A-B) SMINS-MEF-7 neurospheres were cultured in suspending culture (A) and attaching culture (B) under bright field.

(C-D) SMINS-MEF-7 neurospheres were dissociated and stained by typical neural stem cell markers Sox2 (red) and Nestin (red) examined by immunocytochemistry.

(E) Analysis of typical neural stem cell gene expressions by RT-PCR, 1: NS (native neural stem cells), 2: SMINS-TTF-7 (small molecule-induced neural stem cells from TTF with 3 small molecules), 3: MEF (mouse embryonic fibroblasts).

DAPI was used for nuclei counter-staining (blue). Scale bar: 100 μm.

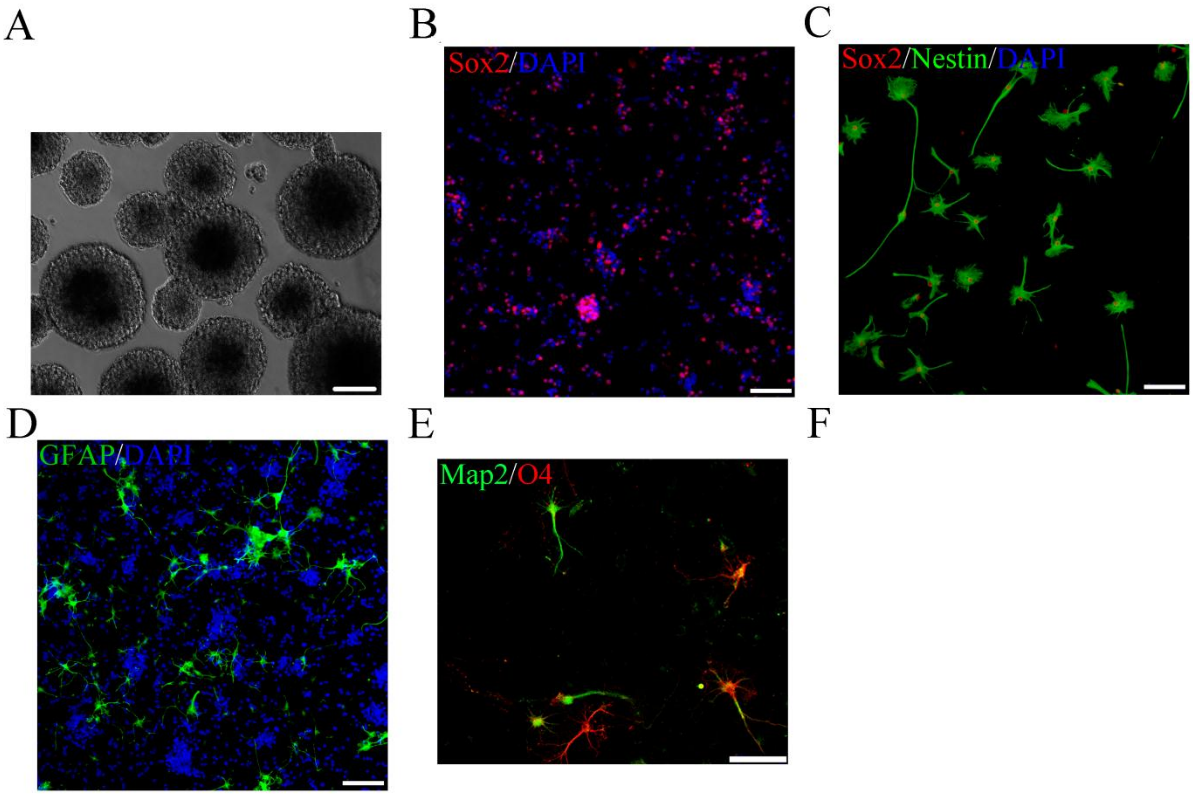

Supplementary Figure S4. The positive control native neural stem cells (NS).

(A) NS cells were cultured in suspending culture under bright field.

(B-C) NS cells were stained by typical neural stem cell markers Sox2 (red) and Nestin (red) examined by immunocytochemistry.

(D-E) NS cells were stained by astrocyte marker GFAP (green), neuron marker Map2 (green) and oligodendrocyte marker O4 (red).

DAPI was used for nuclei counter-staining (blue). Scale bar: 100 μm

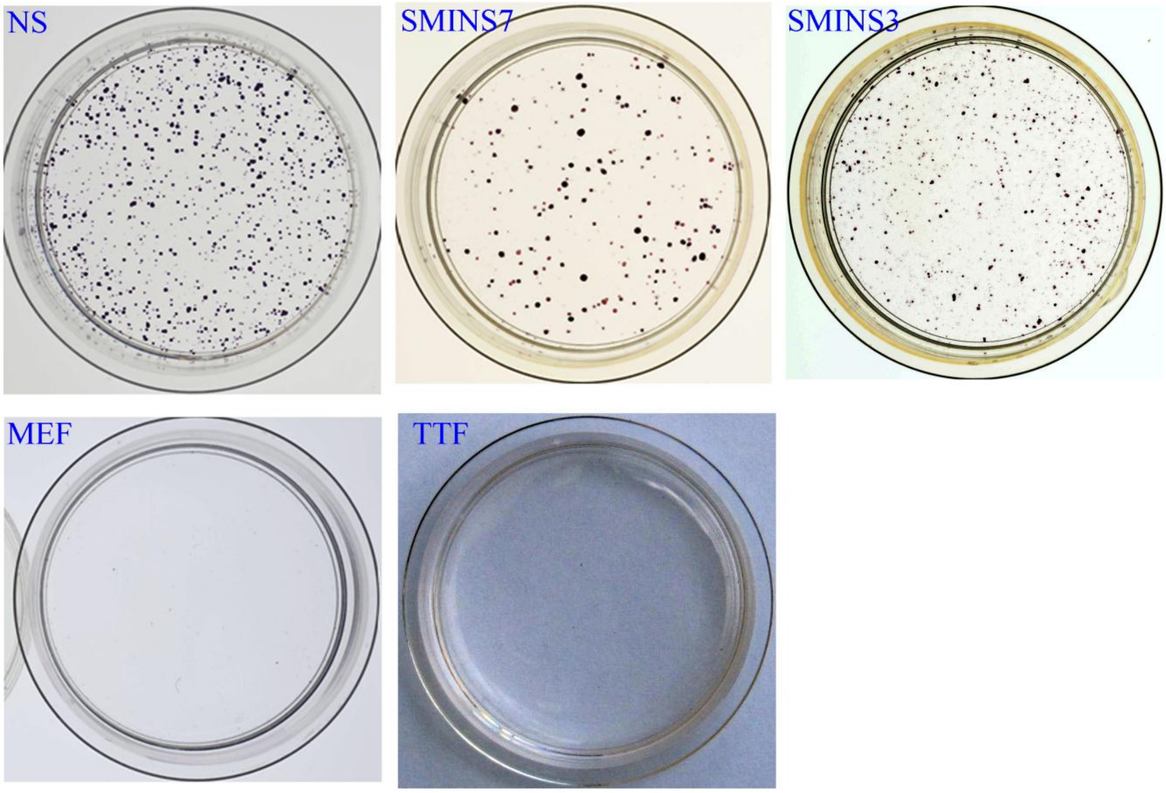

Supplementary Figure S5. The staining of Alkaline Phosphatase (ALP) for NS, SMINS and Fibroblasts.

Positive control neural stem cells, SMINS7 and SMINS3 cells were positive for ALP while MEF and TTF were negative.

A

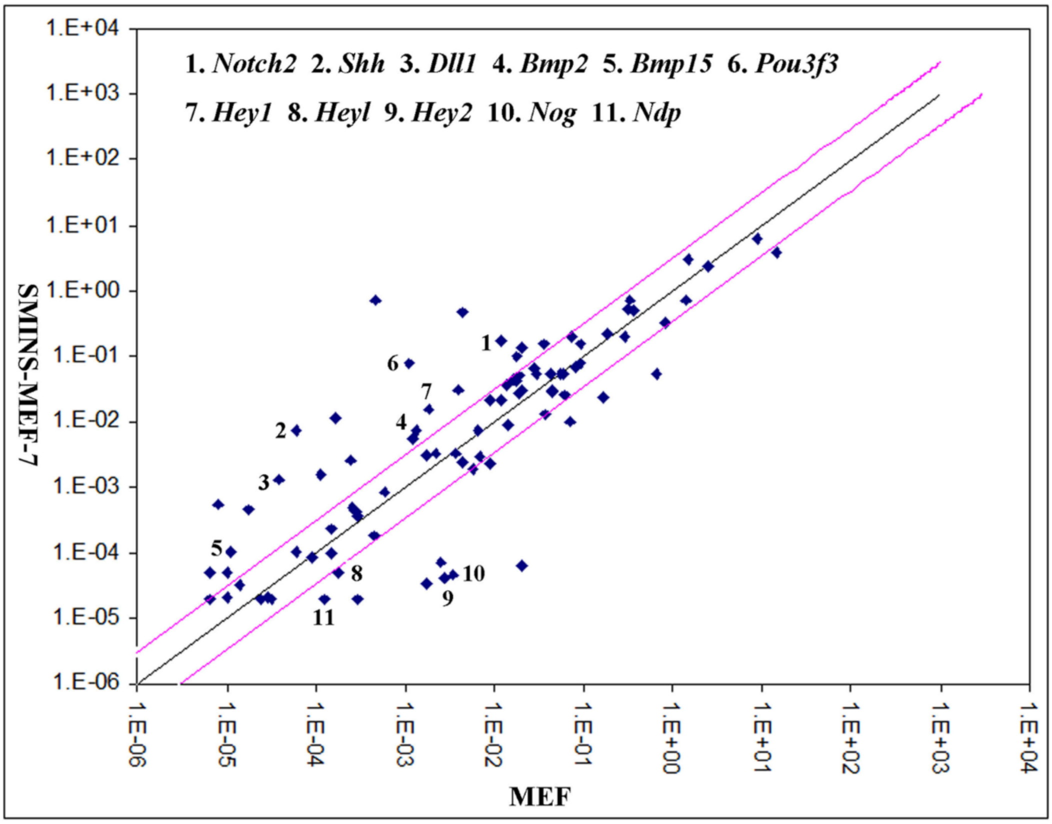
1. Notch2 2. Shh 3. Dll1 4. Bmp2 5. Bmp15 6. Pou3f3
7. Hey1 8. Heyl 9. Hey2 10. Nog 11. Ndp
SMINS-MEF-7
MEF
1.E+04
1.E+03
1.E+02
1.E+01
1.E+00
1.E-01
1.E-02
1.E-03
1.E-04
1.E-05
1.E-06

B

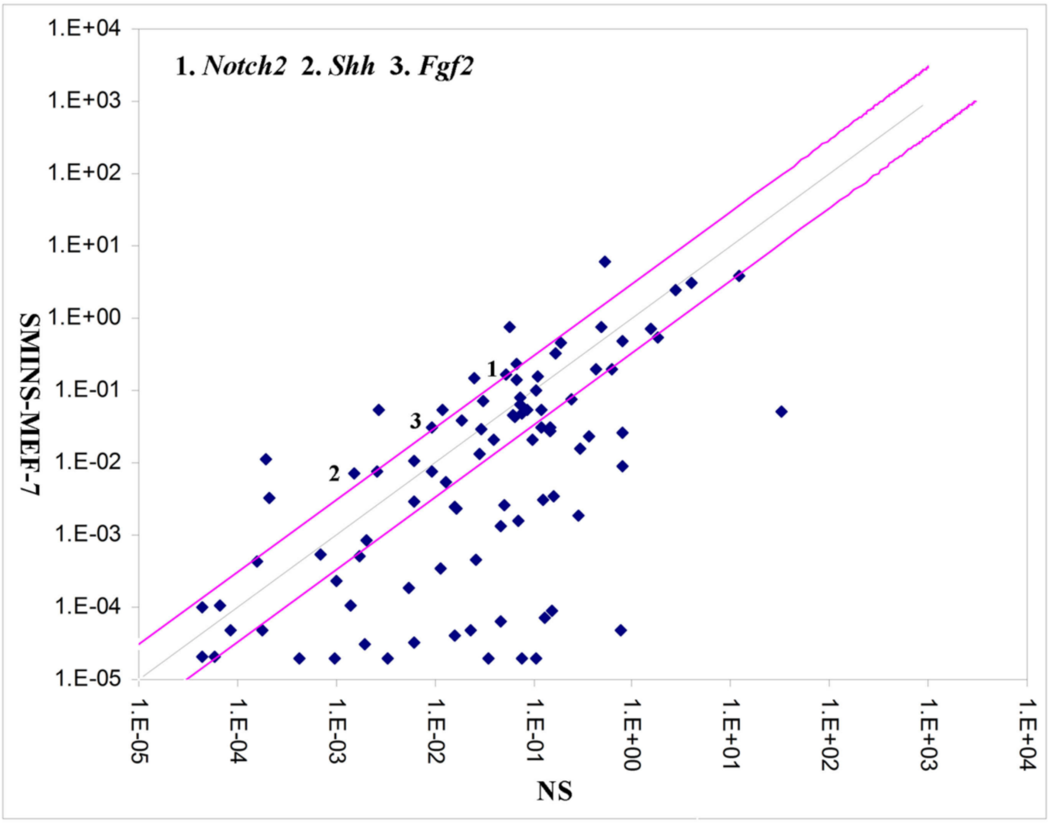
1. Notch2 2. Shh 3. Fgf2
SMINS-MEF-7
NS
1.E+04
1.E+03
1.E+02
1.E+01
1.E+00
1.E-01
1.E-02
1.E-03
1.E-04
1.E-05

Supplementary Figure S6. Quantitative analysis of 84 neural stem cell genes by RT profiler PCR arrays.

(A) SMINS-MEF-7 vs MEF.

(B) SMINS-MEF-7 vs NS. The black line depicts the regression of absolute correlation between two different cell types; the red lines depict the scoring border lines with a two-fold change in standard deviation from the absolute correlation line.

NS: native neural stem cells; SMINS-MEF-7: small molecule-induced neural stem cells from MEF with 7 small molecules; MEF: mouse embryonic fibroblasts.

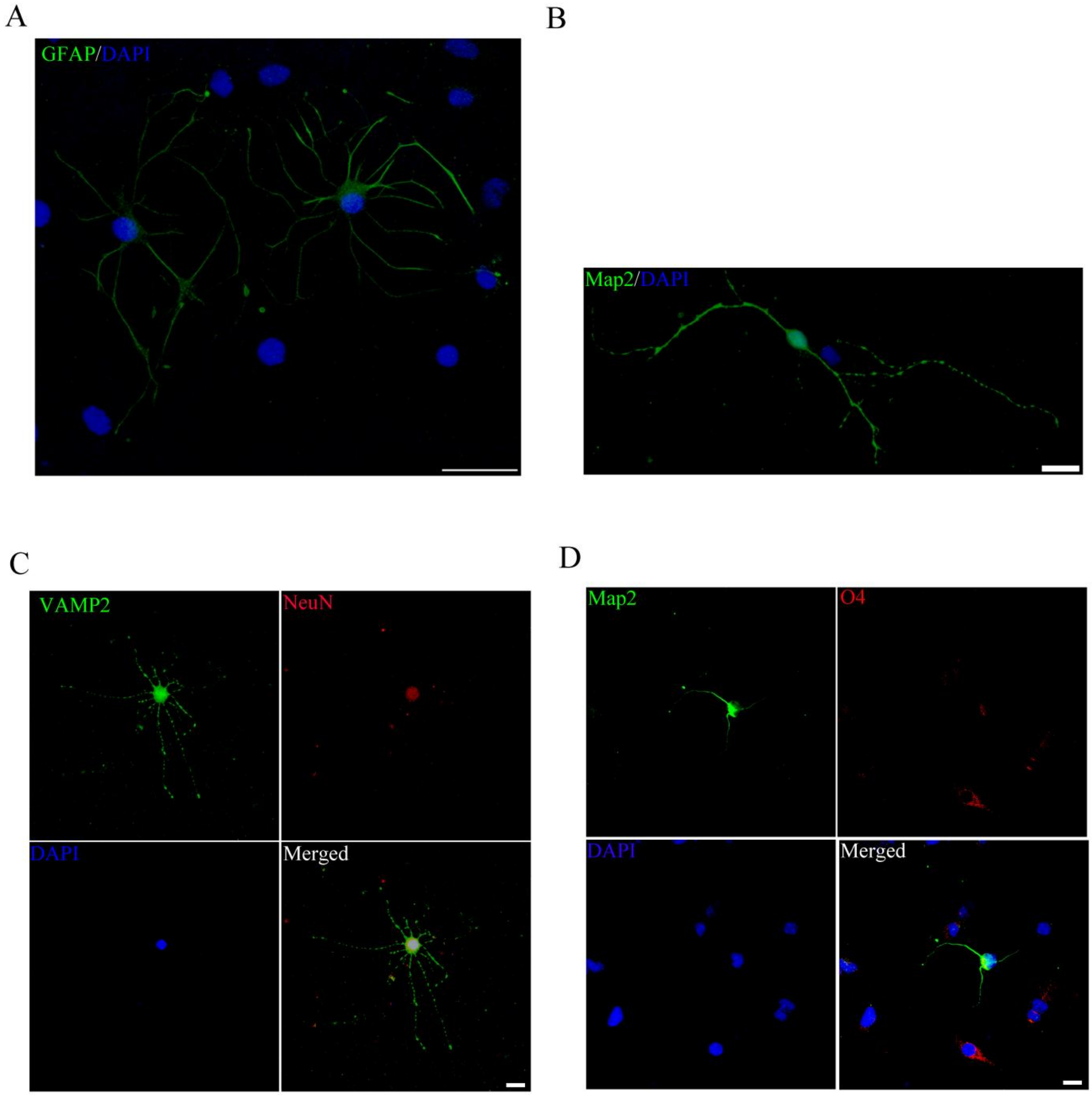
A
GFAP/DAPI
B
Map2/DAPI
C
VAMP2
NeuN
DAPI
Merged
D
Map2
O4
DAPI
Merged

Supplementary Figure S7. Differentiation of SMINS-MEF-7 cells *in vitro*.

(A) SMINS-MEF-7 cells spontaneously differentiated into astrocytes marked by GFAP (green) in spontaneous medium for three weeks.

(B) SMINS-MEF-7 cells spontaneously differentiate into neurons marked by Map2 (green) in spontaneous medium for three weeks.

(C) SMINS-MEF-7 cells were cultured in mature neural solution for one month and stained by mature neuron markers Vamp2 (green) and NeuN (Red).

(D) SMINS-MEF-7 cells spontaneously differentiate into neurons marked by Map2 (green) and oligodendrocyte marker O4 (red) in spontaneous medium for three weeks.

DAPI was used for nuclei counter-staining (blue). Scale bar: 50 µm (A) and 10 µm (B-D).

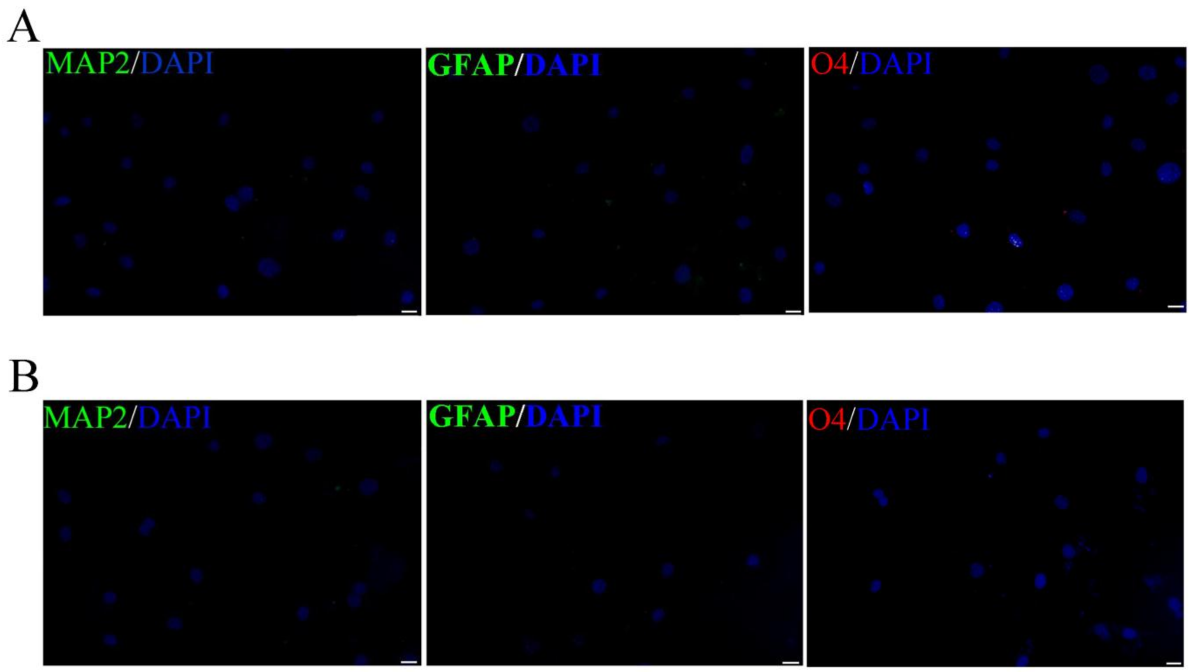

Supplementary Figure S8. Immunocytochemistry staining of fibroblasts using nervous cell markers.

(A) MEF cells were stained by astrocyte marker GFAP (green), neuron marker Map2 (green) and oligodendrocyte marker O4 (red).

(B) TTF cells were stained by astrocyte marker GFAP (green), neuron marker Map2 (green) and oligodendrocyte marker O4 (red).

DAPI was used for nuclei counter-staining (blue). Scale bar: 10 μm.

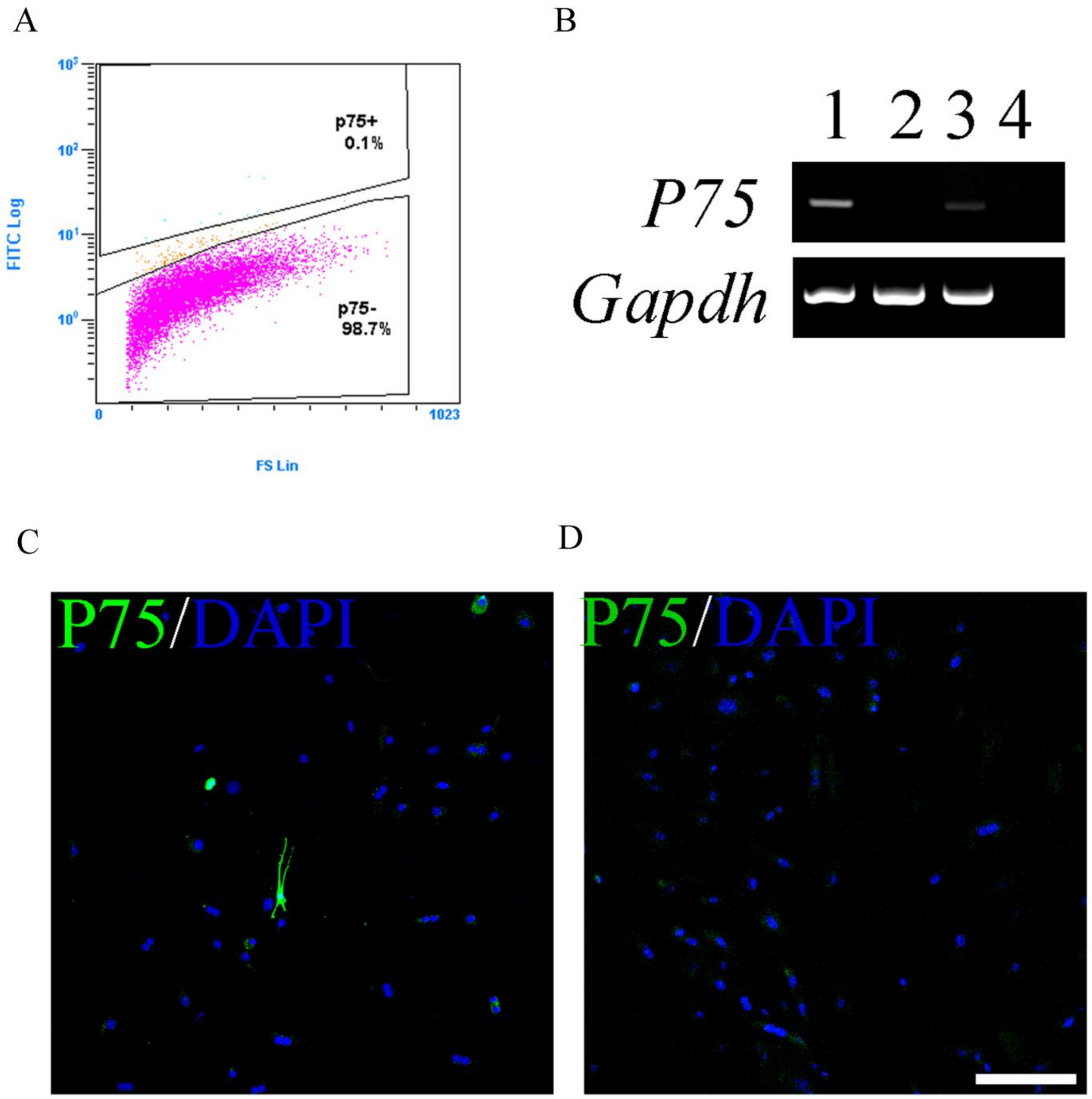

Supplementary Figure S9. The sorting of negative TTF.

(A) TTF cells were sorted by FACS with neural crest marker P75.

(B) RT-PCR for P75 negative TTF cells. 1: NS (native neural stem cells), 2: TTF (Tail-tip fibroblasts) after sorting, 3: TTF, 4: blank.

(C) TTF cells (Passage 3) were stained by neural crest marker P75 (green).

(D) Sorting TTF cells were stained by neural crest marker P75 (green).

DAPI was used for nuclei counter-staining (blue). Scale bar: 100 μm.

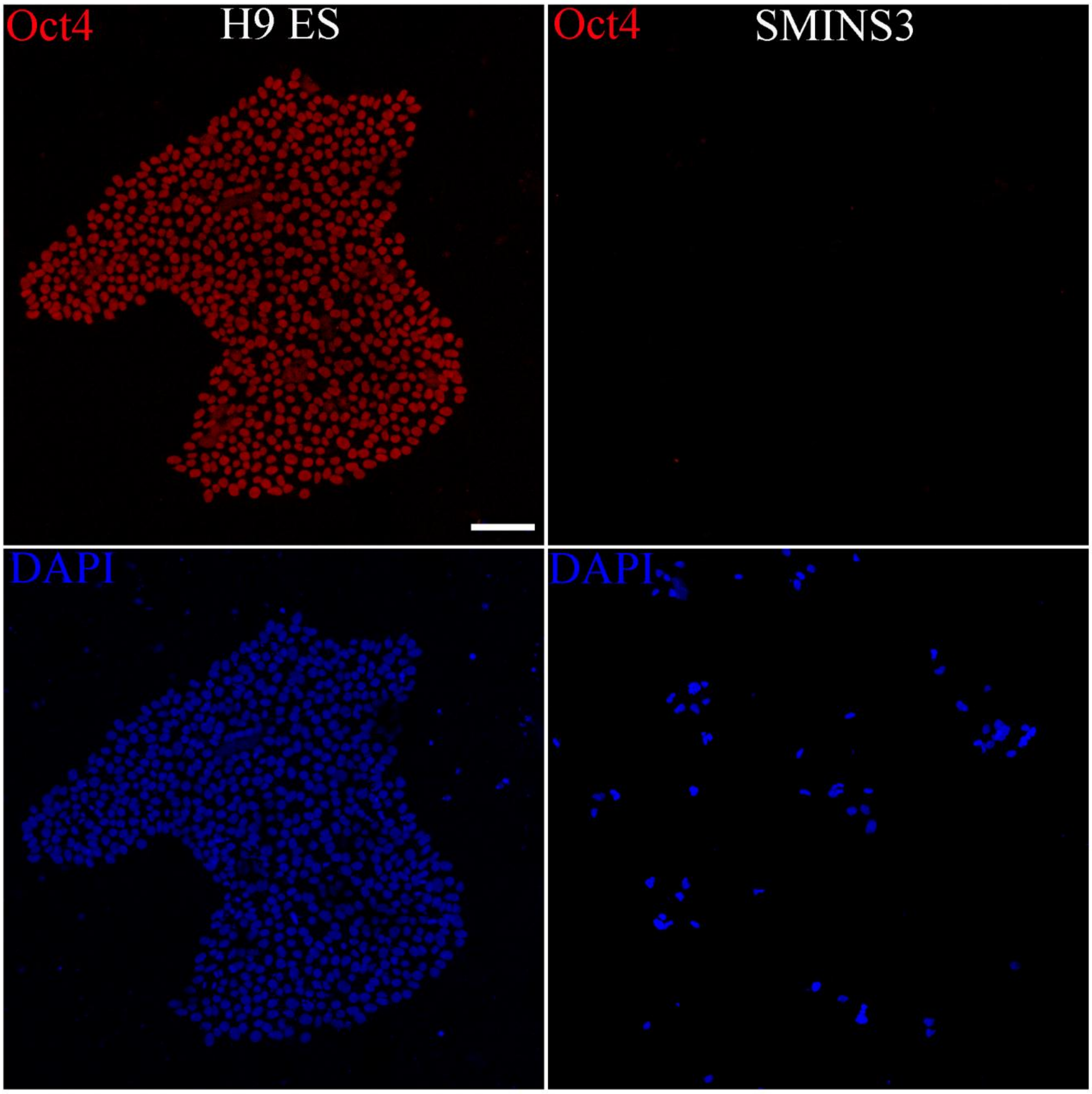

Supplementary Figure S10. SMINS3 cells were check by pluripotent stem cells marker. SMINS3 cells were stained by pluripotent stem cell marker Oct4 and H9 embryonic stem cells were taken as positive control. The reactivity of the antibody Oct4 is for human, rat and mouse.

DAPI was used for nuclei counter-staining (blue). Scale bar: 100 µm.

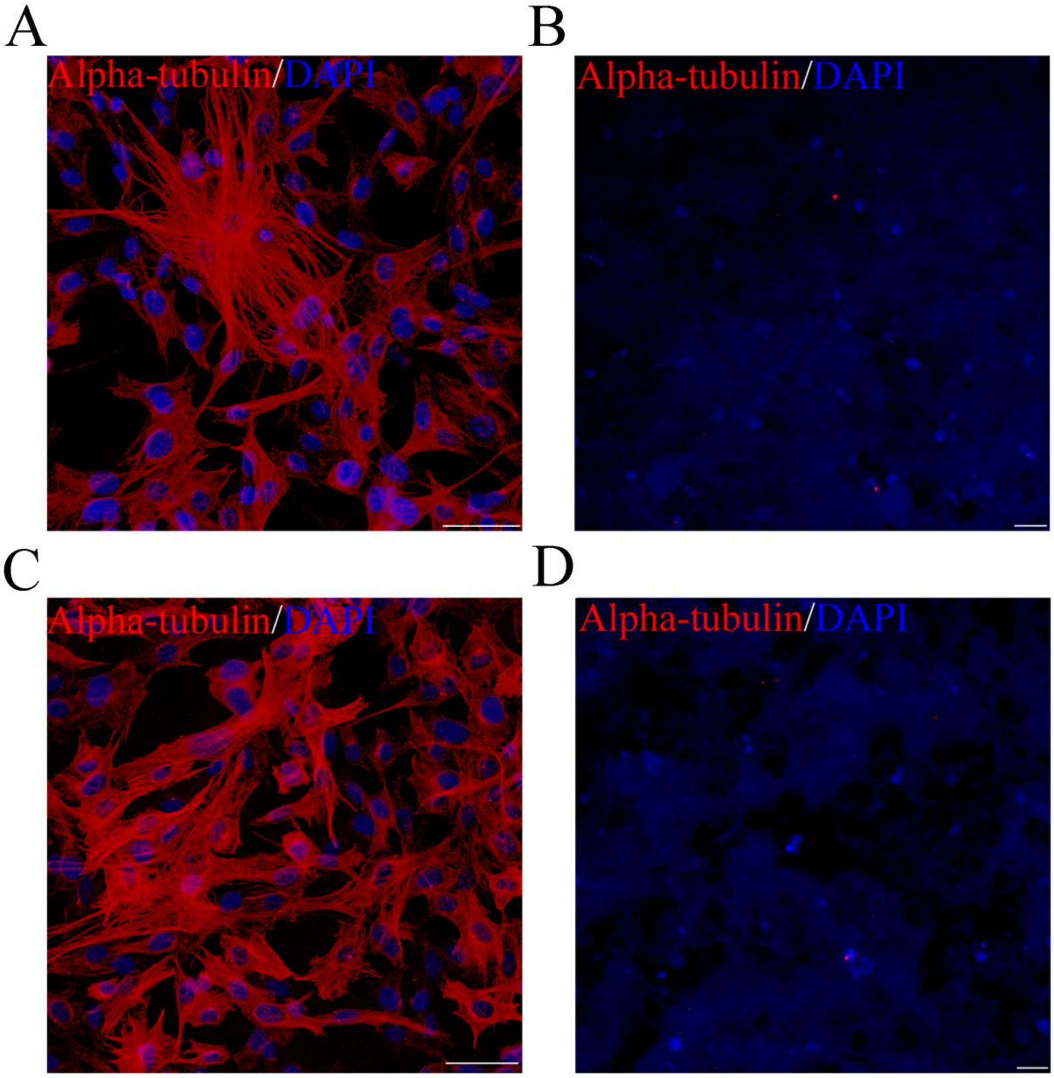

Supplementary Figure S11. Immunocytochemistry staining of fibroblasts and SMINS cells using a fibroblast marker.

(A) MEF cells were stained by Alpha-tubulin (Red).

(B) SMISN-MEF-7 cells after passage 5 were stained by Alpha-tubulin (Red).

(C) TTF cells were stained by Alpha-tubulin (Red).

(D) SMISN-TTF-3 cells after passage 5 were stained by Alpha-tubulin (Red).

MEF: mouse embryonic fibroblasts, TTF: Tail-tip fibroblasts. Scale bar: 50 μm.

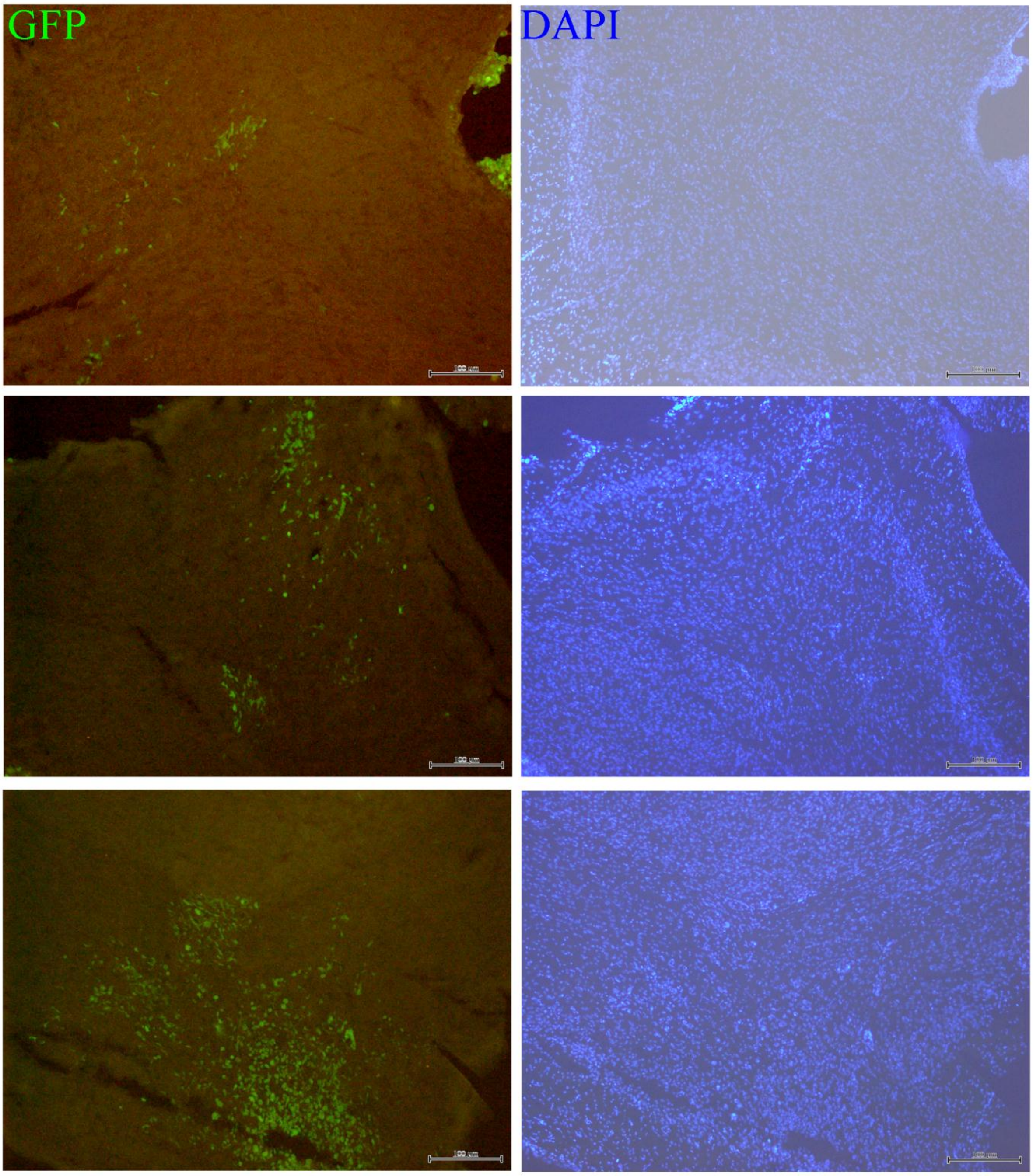

Supplementary Figure S12. Migration of SMINS-TTF-3 cells *in vivo*.

SMINS-TTF-3 cells infected with lentiviral EGFP vectors were injected into the lateral ventricle of brain in nude pups at the age of day 3 and the brains were collected at 6 week point. The injected cells (GFP^+) migrated to other regions of the brain from the injected position.

DAPI was used for nuclei counter-staining (blue). Scale bar: 100 µm.

A B

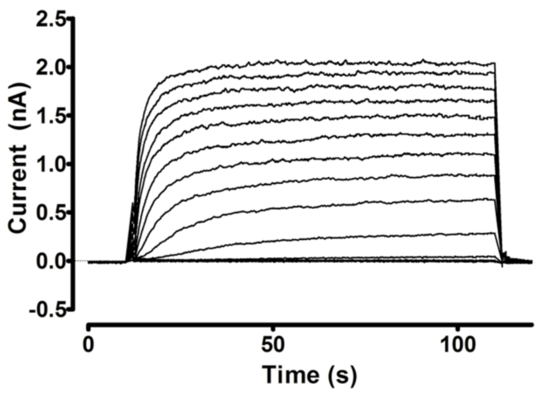

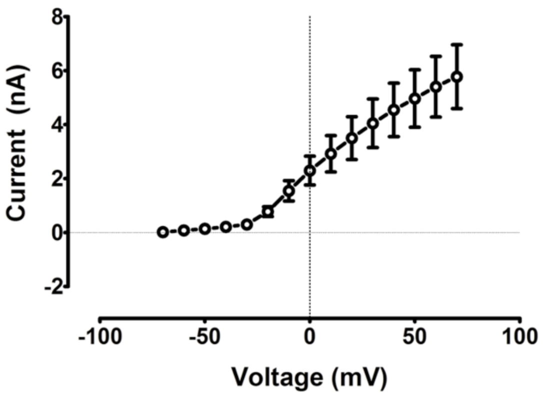

Supplementary Figure S13. The majority of differentiated cells demonstrate K^+ currents only.

Electrophysiological investigations of most differentiated cells showed the presence of only outward K^+ currents in response to electrical stimulation with steps from -70 to +70 mV (10 mV increments) from a holding potential of -80 mV.

(A) Representative traces with 100 ms duration steps.

(B) Mean ± SEM maximal K^+ currents (n = 11 cells).

Supplementary Table S1. Analyses results of up-regulated and down-regulated genes for SMINS-MEF-7 cells (test) vs MEF (control).

Up-regulated genes	Up-regulated fold	Down-regulated genes	Down-regulated fold
Apbb1	7.8	*Fgf13*	329.19
Bmp15	9.29	*Hey2*	67.38
Bmp2	5.62	*Heyl*	3.58
Cdk5rap2	4.40	*Inhba*	12.75
Cxcl1	67.46	*Ndn*	7.14
Dll1	34.20	*Nog*	75.12
Drd2	3.01	*Nrcam*	35.50
Hdac4	5.30	*Pard6b*	3.80
Hey1	8.66	*Pax3*	53.13
Il3	7.50	*Arnt2*	3.13
Neurod1	3.01	*Ascl1*	15.35
Notch2	14.11	*Bdnf*	6.94
Ntn1	107.11	*Ndp*	6.44
Pax6	13.88		
Pou3f3	68.02		
Pou4f1	65.98		
Robo1	6.79		
S100a6	1543.93		
Sema4d	10.06		
Shh	123.47		
Sox3	26.94		
Tnr	4.74		
Vegfa	4.19		

Supplementary Table S2. Analyses results of up-regulated genes for SMINS-MEF-7 cells (test) vs NS (control).

Up-regulated genes	Up-regulated fold
Cxcl1	57.20
Fgf2	3.43
Gdnf	15.62
Inhba	19.22
Notch2	3.23
Nrp1	13.23
S100a6	11.58
Shh	4.90
Slit2	4.52
Vegfa	6.51

Supplementary Table S3. The primers of RT-PCR in the experiment.

Gene	Forward	Reverse
Olig2	TCATCTTCCTCCAGCACCTC	CCGTAGATCTCGCTCACCAG
Gli2	GCCTGAGCCTTTGCCTTCAC	ATGTTGCTGATGCCCGCAG
GFAP	ACCATTCCTGTACAGACTTTCTCC	AGTCTTTACCACGATGTTCCTCTT
Oct4	CCAACGAGAAGAGTATGAGGC	CAAAATGATGAGTGACAGACAGG
Sox2	TCTGTGGTCAAGTCCGAGGC	TTCTCCAGTTCGCAGTCCAG
Nanog	CCTCCAGCAGATGCAAGAA	GCTTGCACTTCATCCTTTGG
Gapdh	ACCACAGTCCATGCCATCAC	TCCACCACCCTGTTGCTGTA
